# Pulmonary flow profile and distensibility following acute pulmonary embolism

**DOI:** 10.1186/1532-429X-13-14

**Published:** 2011-02-18

**Authors:** Frederikus A Klok, Soha Romeih, Jos JM Westenberg, Lucia JM Kroft, Menno V Huisman, Albert de Roos

**Affiliations:** 1 Section of Vascular medicine, department of General Internal Medicine-Endocrinology, LUMC, Leiden, the Netherlands; 2 Department of Radiology, LUMC, Leiden, the Netherlands

## Abstract

**Objective:**

Proof of concept study evaluating CMR as screening tool for chronic thromboembolic pulmonary hypertension (CTEPH) in patients treated for acute pulmonary embolism (PE).

**Materials and methods:**

Right and left ventricular function of 15 consecutive patients treated for PE and 10 consecutive patients in whom PE was excluded was estimated at baseline by cardiac CT and at 6 months follow-up by CMR. Additionally, during the follow-up visit, pulmonary artery (PA) hemodynamics were studied by CMR and the presence of pulmonary hypertension by echocardiography.

**Results:**

CT measured right ventricular ejection fraction (RVEF) was lower in patients with PE compared to patients without PE at time of diagnosis (median 47%, interquartile range 39-53 vs. 55%, 52-58; p = 0.014). After 6 months follow up, the RVEF between patients treated for PE and patients without PE were not statistically significant different (55%, 52-60 versus 54%, 51-57; p = 0.57), as were distensibility index (0.18 ± 0.18 versus 0.25 ± 0.18, p = 0.20), mean velocity (14.1 ± 3.9 cm/s versus 14.0 ± 2.5 cm/s, p = 0.81), peak velocity (86.5 ± 22 cm/s versus 89.6 ± 13 cm/s, p = 0.43) and time to peak PA blood flow velocity (142 ± 49 ms versus 161 ± 29 ms, p = 0.14). One patient was diagnosed with CTEPH and CMR revealed poor right systolic function, decreased PA distensibility and flow velocity, and a systolic notch in the PA flow profile consistent with persistent PA obstruction.

**Conclusion:**

In this small series, right ventricular performance and PA flow profiles of patients treated for 6 months after PE are equivalent to those parameters in normal patients.

## Introduction

Acute right ventricular (RV) dysfunction associated with pulmonary embolism (PE), which can both be evaluated by multi-detector row CT, is caused by increased tension in the RV wall and may lead to RV dilatation and ischemia [1,2]. The natural history of RV recovery after acute PE is largely unknown. Persistent RV dysfunction after PE might be a predictor of chronic thromboembolic pulmonary hypertension (CTEPH), a rare but serious long term clinical complication of PE [3]. Because the underlying pathophysiological mechanism leading to CTEPH is not fully established and its clinical presentation is not specific, the early identification of patients with CTEPH is very difficult [3]. Consequently, the majority of CTEPH patients present with more advanced stage disease.

The reference standard for diagnosing pulmonary hypertension is right heart catheterization [4]. Currently, the most widely used non-invasive screening tool for pulmonary hypertension is echocardiography, although it has been shown that Doppler echocardiography may frequently be inaccurate in estimating pulmonary artery pressure and cardiac output in patients being evaluated for pulmonary hypertension [5]. Cardiovascular magnetic resonance (CMR) is a non-invasive modality for evaluating pulmonary hypertension by evaluation of left and right systolic and diastolic function as well as by quantification of pulmonary artery distensibility and pulmonary flow dynamics [6-10]. CMR measured pulmonary flow dynamics are altered in acute PE as well as in pulmonary hypertension, and have been shown to correlate closely with invasive assessment of cardiac hemodynamic function and clinical outcome in patients with pulmonary hypertension [6-10]. Especially pulmonary artery distensibility has been suggested to be a sensitive (sensitivity 83%) and specific (specificity 82%) marker for pulmonary hypertension, even in early or mild clinical stages [9,10].

We hypothesized that the pulmonary flow dynamics would restore to normal after 6 months of treatment following acute PE and therefore would not be different from patients without PE, except for those patients who develop CTEPH who will show decreased distensibility and flow velocity in the pulmonary artery. Following this, flow profile analysis of the pulmonary artery in the clinical follow-up of patients with acute PE might be a helpful screening tool for CTEPH. Accordingly, we performed a proof of concept study to evaluate pulmonary artery hemodynamics and distensibility at 6 months follow-up in consecutive patients with PE, and in patients in whom PE was clinically suspected but ruled out as a control cohort.

## Materials and methods

### Patients

Since this was a proof of concept study to evaluate the restoration of pulmonary artery hemodynamics, we aimed at studying 15 consecutive patients treated for and 10 consecutive patients in whom PE was ruled out. Consecutive, hemodynamically stable in- and outpatients suspected of acute PE were eligible. All patients underwent multi-detector row computed tomography (MDCT) of the chest to establish or rule out acute PE as described by Huisman et al [11]. The presence of PE was defined as at least one filling defect in the pulmonary artery tree. Furthermore, in all patients, a separate image acquisition using retrospective ECG-gated dynamic cardiac MDCT was performed to assess right and left ventricular function. All scans were performed according to a standardized protocol, described previously by Dogan et al in full detail [2]. Scan parameters were: tube voltage 120 kV and tube current 200 mA. The optimal pitch factor and rotation time were automatically established to obtain optimal temporal resolution. Images for functional analysis were reconstructed in 20 cardiac phases by using a segmental reconstruction algorithm. The entire heart from aortic root to cardiac apex was covered within the reconstructed sections per cardiac phase point. The reconstructed volumes were transferred to a dedicated workstation running on Linux software.

ECG-gated multi-detector computed tomography (MDCT) has been shown to be a reliable method to assess ventricular volumes and ejection fraction and can be combined with computed tomography pulmonary angiography to establish the diagnosis of PE [2,11-14]. Finally, the severity of the pulmonary obstruction was measured following the method described by Qanadli and colleagues [15]. Patients with confirmed PE were initially treated with therapeutic unfractioned or low-molecular-weight heparin, followed by vitamin K antagonists for 6 months [16]. Study participants were excluded if they had a contraindication for CMR scanning, e.g. pregnancy, aneurysm clip in the brain, implanted neural stimulator, implanted cardiac pacemaker or defibrillator, or severe claustrophobia. The study was approved by an institutional review board and all participants consented to participation.

### Follow-up CMR

After 6 months following initial presentation, CMR examinations were performed using a 1.5T CMR scanner (ACS-NT15 Intera, Philips Medical Systems, Best, the Netherlands). We used a five-element phased-array cardiac coil placed on the chest for signal reception. First, a stack of 14-18 transverse slices (dependent on the size of the heart) was achieved during breath-holding at end-expiration and by using a steady-state free-precession sequence for biventricular volume measurements. We used the following scan parameters: slice thickness 10 mm with no gap, field-of-view = 450 mm (80% rectangular), scan matrix = 256 × 195, with reconstructed voxels of 1.37 × 1.37 × 8.0 mm, flip angle α = 35°, repetition time (TR) 3.2 ms and echo time (TE) 1.6 ms. We utilized gated cardiac synchronization (30 reconstructed phases per cardiac cycle, temporal resolution 20-35 ms) and parallel imaging (Sensitivity encoding SENSE, sense factor 2). Using the MASS software package, we drew the endocardial contours at end-systole and end-diastole manually.

Second, the main pulmonary artery flow curve was obtained using velocity-encoded (VE) CMR, planned perpendicular to the pulmonary artery. The VE CMR acquisition was performed during breath-holding, with the acquisition plane planned perpendicular to the pulmonary trunk distal to the pulmonary valve. Scan parameters: slice thickness 8 mm; field-of-view = 300 mm (85% rectangular), scan matrix = 128 × 108, with reconstructed voxels of 1.17 × 1.17 × 8.0 mm, flip angle α 20°, TR/TE = 9.3 ms/6.1 ms, two signal averages, VENC = 100 cm/s with echo planar imaging (EPI) factor 7. Pulmonary artery time-to-peak-velocity, peak-velocity and mean velocity were assessed. Pulmonary artery contours were semi-automatically drawn using the FLOW software package (FLOW software package; Medis, Leiden, The Netherlands).

Distensibility index [(Area max (systole) - Area min (diastole))/(Area min)] was determined from the lumen area measurements of the pulmonary artery at the moment of maximal flow and at the moment of the isovolumetric contraction [7]. Two gated acquisitions were performed to obtain the maximal and minimal luminal areas, using steady-state free precession sequences with a field-of-view of 220 mm, voxel size 1.25 × 1.25 × 6.00 mm, flip angle 50°; TR/TE 3.2/1.2 and a gate width of 34.2 ms. The gate delay was accordingly set that the middle of the acquisition window equals the moment of maximal flow or the moment of isovolumetric contraction, respectively. Also, in order to correct for through-plane motion of the acquisition plane, the location and angulation of this plane were manually adjusted on both orthogonal cine views of the right ventricular outflow tract, specifically on the two phases of the cardiac cycle nearest to the chosen gate delays as described by Grotenhuis et al [17].

All contours were drawn by one observer (2 years experience with CMR) supervised by a radiologist (11 years experience with CMR) who were both blinded for the patients' condition.

### Echocardiography

To evaluate the presence of pulmonary hypertension in the study patients, all underwent transthoracic echocardiography after the CMR was performed. Echocardiography included cross sectional, M-mode and Doppler studies, and was performed by an experienced technician according to a standardized protocol. In case of suspected pulmonary hypertension (1) maximal tricuspid regurgitation velocity >2.8 m/s, 2) estimated systolic pulmonary artery pressure ≥35 mmHg, 3) estimated mean pulmonary artery pressure ≥25 mmHg 4) borderline value of criterion 1 or 2 in combination with a right ventricular TEI index >0.36 [18]) or other echocardiographic abnormalities and if clinically indicated, further diagnostic work-up was performed under supervision of an independent expert panel. Criteria for the diagnosis of CTEPH were mean pulmonary artery pressures assessed by right heart catheterization exceeding 25 mmHg respectively and normal pulmonary capillary wedge pressure in combination with an abnormal perfusion scintigram and signs for distal or central CTEPH on conventional pulmonary angiography [4].

### Statistical analysis

Differences in baseline characteristics and CMR measurements between patients with and without PE were sought for using the Student's T-test in case of normal distribution or else the Mann-Whitney U test for pairwise comparisons. Variables that were normally distributed are presented as mean and standard deviation, variables with skewed distribution as medians and interquartile range. The presence of normal distribution was tested using the Kolmogorov-Smirnov test. Finally, we compared the CMR test results of patients with to those without pulmonary hypertension. P-values less than 0.05 were considered significant.

## Results

### Study patients

To achieve our sample size goal, we followed 27 consecutive patients with acute PE and 15 consecutive patients in whom PE was ruled out. From these patients, 3 had died during the 6 months follow-up period and 14 were excluded because of implanted cardiac pacemaker, unwillingness to cooperate or claustrophobia, leaving 15 patients diagnosed with and 10 patients without PE for analysis. The demographic characteristics of the two patient cohorts were comparable (table 1); overall mean age was 53 ± 11 years and 14 (60%) of the patients were of male gender. The distribution of cardiopulmonary comorbidity was comparable as well. None of the patients without PE had a history of venous thrombosis or developed venous thrombosis during the 6 months follow-up period. Median follow-up duration of the overall population was 205 days (range 165-301 days), and was not different between the two study cohorts (table 1).

**Table 1:** Baseline characteristics of study patients

	PE patients (n = 15)	Patients without PE (n = 10)	
Age ([years] mean, ± SD)	53 ± 10	52 ± 13	NS

Female gender (n, %)	7 (47)	7 (70)	NS

History of venous thrombosis (n, %)	6 (40)	0 (0)	NS

COPD (n, %)	3 (20)	2 (20)	NS

Pre-existent left heart failure (n, %)	1 (6.7)	0 (0)	NS

Qanadli score (median, IC range)	15 (2-26)	NA	

Right ventricular ejection fraction by CT (%, median, IC range)	47 (39-53)	55 (52-58)	p = 0.014

Left ventricular ejection fraction by CT (%, median, IC range)	54 (31-63)	60 (55-65)	p = 0.038

Follow-up duration (days; mean, ± SD)	226 ± 42	202 ± 36	NS

PE = pulmonary embolism, n = number, SD = standard deviation, NA = not applicable, IC = interquartile

The median Qanadli score of the patients with PE was 15 with a range of 2 to 26 (table 1). The systolic performance of both the right and left ventricle of the patients with PE was significantly impaired compared to the control patients without PE: median right ventricular ejection fraction 47 (39-53) versus 55 (52-58; p = 0.014) and mean left ventricular ejection fraction 54.1 ± 8.2% versus 60.1 ± 3.5% (p = 0.038) for patients with and without PE respectively.

### Pulmonary artery flow dynamics

**Figure 1:**
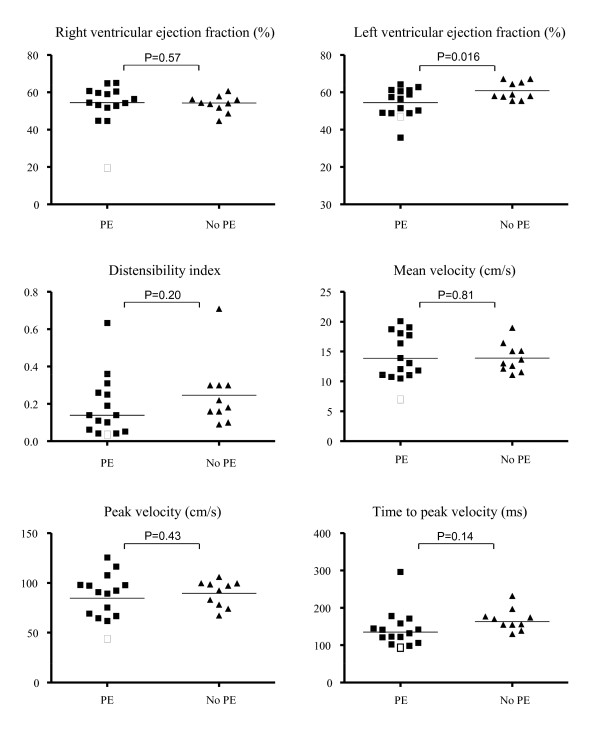
**Distribution of right and left ventricular ejection fraction, distensibility index and flow characteristics of the pulmonary artery in the study population**. The patient with CTEPH is indicated with the open box. Medians (right ventricular ejection fraction) and means (all other parameters) are indicated. *p < 0.05

After six months follow up, right ventricular ejection fraction was not statistically significant different between patients with PE and control patients without PE (median 54.5%; interquartile range 51.8-60.4 versus 54.3%; 51.0-56.6, p = 0.57, Figure 1). In contrast, patients with PE had statistically significant lower left ventricular ejection fraction than patients without PE (mean 54.7% ± 5.8 versus 59.5% ± 3.5, p = 0.016). The pulmonary artery distensibility index was not different between patients with and without PE (0.18 ± 0.18 versus 0.25 ± 0.18, p = 0.20). Also, the studied pulmonary hemodynamic parameters were not different between patients with and without PE: mean velocity 14.1 ± 3.9 cm/s versus 14.0 ± 2.5 cm/s (p = 0.81), peak velocity 86.5 ± 22 cm/s versus 89.6 ± 13 cm/s (p = 0.43), and time to peak velocity 142 ± 49 ms vs 161 ± 29 ms (p = 0.14; Figure 1).

### CMR as potential screening tool for CTEPH

**Figure 2:**
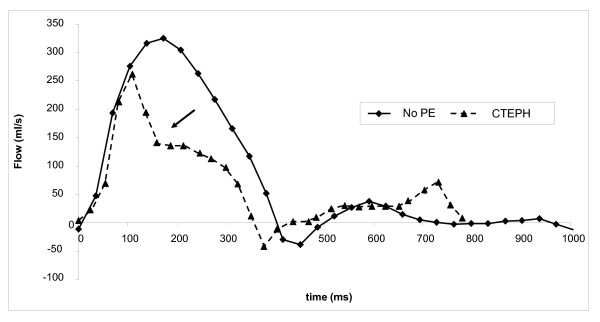
**Pulmonary artery flow curve of the patient that was diagnosed with CTEPH and that of a healthy control without PE**. Note the pulmonary flow systolic notch (arrow) and also diastolic forward flow as marker of restrictive physiology

All study participants underwent echocardiography after the CMR scan that revealed only one patient with PE suspected of having pulmonary hypertension. The diagnosis of inoperable CTEPH was confirmed after right heart catheterization and conventional angiography. This female patient was 59 years old, was diagnosed initially with idiopathic pulmonary embolism and expressed symptoms of exertional dyspnea and decreased exercise tolerance. At time of diagnosis of CTEPH, she was classified in NYHA class III and her mean pulmonary arterial pressure was 48 mmHg. Results from the CMR measurements in this patient indicated decreased systolic performance and increased stiffness of the pulmonary artery (Figure 1). Even more, she had the lowest right ventricular ejection fraction (19.9%), distensibility index (0.03), mean pulmonary artery velocity (7.11 cm/s), peak pulmonary artery velocity (44.6 cm/s) and time to peak velocity (97 ms) of all study patients. Strikingly, the pulmonary artery flow curve of the patient with CTEPH had an abnormal shape, i.e. a steep pulmonary flow systolic notch (Figure 2). This notch represents the increased wave reflection in the pulmonary artery caused by a stiffened pulmonary artery wall or obstruction of the blood flow, and the timing of the notch distinguishes proximal from distal obstruction of the pulmonary artery in acute PE as well as in CTEPH [19].

## Discussion

The main finding of this study is that CMR measured pulmonary artery flow profiles of patients treated for acute PE do not differ from that of control patients without PE, whereas the same patients with PE had significantly different systolic cardiac performance compared to the same control patients without PE at time of diagnosis before treatment was initiated. This observation possibly indicates normalization of the pulmonary flow dynamics in the majority of patients after PE. One additional finding of this study is that CMR is a potentially valuable screening tool for CTEPH after PE since measurements of the pulmonary artery flow profile in combination with RV function might be discriminative for pulmonary hypertension.

There is great need to develop tools for early identification of patients in early clinical stages of CTEPH. Only in case of successful pulmonary endarterectomy, CTEPH is a potentially curable but otherwise lethal disease [2]. Early identification of CTEPH is likely to improve the disease specific prognosis since even when pulmonary endarterectomy is achievable, pulmonary artery pressure and resistance as well as functional status of the patients remain important prognostic factors [20]. Because the incidence of CTEPH is reported to be as high as 3.8% or even 8.8% [21,22], screening programs for CTEPH might be considered in the clinical follow-up of patients with acute PE. Such screening programs should employ tools that are non-invasive, widely available and applicable, and importantly, can distinguish patients who are in early stages of CTEPH from those who are not at risk of developing this condition. The results of this study support the potential role of CMR as early screening tool for CTEPH after acute PE for two reasons. CMR is a widely available and non invasive imaging modality. Furthermore, since the hemodynamics of the pulmonary artery are restored after 6 months of treatment for acute PE, and patients with CTEPH have a clearly different flow profile [6-10], CMR may be able to distinguish patients with clinical relevant pulmonary hypertension from those who are fully recovered, although the design and the number of cases with pulmonary hypertension in our study does not allow to access the value of CMR as a screening tool.

By design of our study, we were not able to evaluate the ability of CMR to identify patients with very early stages of disease, who are likely to develop symptomatic CTEPH over time. Nonetheless, previous reports have suggested that pulmonary artery distensibility is decreased during acute PE, but recovers over time after treatment [9]. Furthermore, pulmonary artery distensibility increases early in the course of pulmonary hypertension, i.e. even when pulmonary hypertension is detectable only with exercise and before overt pressure elevations occur at rest [10]. These reports as our results underline the potential of CMR as screening tool for CTEPH.

The strengths of our study include the prospective design and the inclusion of consecutive patients. We performed well validated CMR scanning protocols and previous studies indicate excellent reproducibility for the used methods [19]. In addition to accurate measurements of cardiac volumes, CMR is widely accepted as the reference standard for evaluating the pulmonary artery flow. Study limitations are the limited sample size and only six months follow up without pulmonary flow measurements at the time of the acute event, which would have allowed evaluating the usefulness of CMR as early screening tool for CTEPH and possible changes of pulmonary flow profiles over time. Future studies should include larger patient cohorts and longer follow-up period. Furthermore, focus of these studies should not only be to detect patients with overt CTEPH but also on establishing relevant threshold values for pulmonary hemodynamic parameters to indentify patients who are at risk of developing clinical relevant CTEPH in the following years. These patients then could be subjected to intensified clinical surveillance or referred to specialized pulmonary hypertension centers, to facilitate early diagnosis and treatment, leading to improved prognosis.

In summary, right ventricular systolic performance and flow curve profiles of patients with PE after 6 months of treatment are generally comparable to those in patients in whom PE was suspected but ruled out. Furthermore, CMR may be helpful to identify patients with CTEPH. Further studies are needed to evaluate the potential role of CMR as screening tool for CTEPH after acute PE.

## Competing interests

The authors declare that they have no competing interests.

## Authors' contributions

FAK was responsible for study concept and design, acquired, analyzed, and interpreted the data,

and drafted the manuscript, he takes responsibility for the integrity of the work; SAHR and JJMW were responsible for study concept and design, acquiring and interpretation of the data and critically revised the manuscript for important intellectual content, LJMK was responsible for data analysis and interpretation and critically revised the manuscript for important intellectual content; MVH and AR were responsible for study concept and design and critically revised the manuscript for important intellectual content. All authors read and approved the final manuscript.
